# The psychological subtype of intimate partner violence and its effect on mental health: protocol for a systematic review and meta-analysis

**DOI:** 10.1186/s13643-019-1118-1

**Published:** 2019-08-09

**Authors:** Sarah Dokkedahl, Robin Niels Kok, Siobhan Murphy, Trine Rønde Kristensen, Ditte Bech-Hansen, Ask Elklit

**Affiliations:** 1 National Centre of Psychotraumatology, Department of Psychology at the University of Southern Denmark, 5230 Odense M, Denmark; 2 Department of Psychology at the University of Southern Denmark, Odense, Denmark; 3 Centre for Innovative Medical Technology, Odense University Hospital, Odense, Denmark; 4 Centre for persons subjected to violence, Frederiksberg Hospital, Frederiksberg, Denmark

**Keywords:** Psychological violence, Aggression, Coercion, Emotional abuse, Intimate partner violence, Mental health, PTSD, Psychometrics, Assessment, Abbreviations, DSM Diagnostic and statistical manual of mental disorders, EIGE European Institute of Gender Equality, ICD International Classification of Diseases, IPV Intimate Partner Violence, NICE National Institute for Health and Care Excellence, PMWI Psychological Maltreatment of Women Inventory, PTSD Posttraumatic Stress Disorder

## Abstract

**Background/aim:**

Psychological violence is estimated to be the most common form of intimate partner violence (IPV). Despite this, research on the independent effect of psychological violence on mental health is scarce. Moreover, the lack of a clear and consistent definition of psychological violence has made results difficult to compare. The present study therefore aims to consolidate knowledge on psychological violence by conducting a systematic review and random-effects meta-analysis on the association between psychological violence and mental health problems, when controlling for other types of violence (e.g. physical and sexual) and taking into account severity, frequency, and duration of psychological violence.

**Method:**

The present study is registered in the International Prospective Register for Systematic Reviews (PROSPERO; #CRD42018116026) and the study design follows the Preferred Reporting Items for Systematic Reviews and Meta-Analyses (PRISMA; Additional file 1). A dual search will be conducted in the electronic databases PsycINFO, PubMed, EMBASE, and Web of Science. Data will be extracted using Endnote and Covidence and a meta-analysis will be conducted using Metafor-package in the programming language R. The Quality Assessment Tool for Quantitative Studies developed by the Effective Public Health Practice Project will be used to assess the quality of the included studies (i.e. weak, moderate and strong).

**Results and discussion:**

The present review will help consolidate knowledge on psychological violence by evaluating whether frequency, severity or actual “type” of psychological violence produces the most harm. A thorough quality assessment will help overcome potential limitations regarding expected variations in terminology and assessment of psychological violence.

**Systematic review registration:**

PROSPERO CRD42018116026.

**Electronic supplementary material:**

The online version of this article (10.1186/s13643-019-1118-1) contains supplementary material, which is available to authorized users.

## Background

Intimate partner violence (IPV) is a global health problem characterized as any behaviour within an intimate relationship that causes physical, psychological or sexual harm [1]. At present, it is well-documented that IPV can cause extensive mental health consequences among its victims [2–5]. IPV can be characterized as an interpersonal trauma, and symptoms of posttraumatic stress disorder (PTSD) have been identified in 31–84.4% of women exposed to IPV [2]; along with other comorbid symptoms such as depression, anxiety, suicidality, substance abuse and sleep disturbances [2, 5, 6].

The subtype of psychological violence (compared to physical and sexual violence) is estimated to be the most common form of IPV in both the USA [7] and Europe [8], affecting between 35 and 49% of men and women. This has led legislators in some European countries to criminalize psychological violence as an independent offence, making it equally punishable as physical violence (e.g. Norway [9] and England [10]). Although some researchers have argued that psychological violence in itself cannot be classified as a trauma, as it does not meet the first criterion of diagnosing PTSD (i.e. threat to life or physical integrity [11, 12]), a more recent review on IPV and mental health argues that psychological violence can independently cause PTSD, depression and anxiety [13].

Despite both legal recognition of psychological violence and documentation of its effect on mental health, the conceptualization of the phenomenon is ambiguous in both research and clinical practice. Acts of psychological violence are distributed along a continuum starting from what is commonly termed *psychological aggression* (e.g. yelling and insults) and ending with more severe abuse, often labelled *coercion* (e.g. threats and isolation). How we interpret *psychological aggression* and how we distinguish it from more severe abuse depends, among other things, on the context in which it occurs, when it occurred in a sequence, how it was interpreted and whether it was perceived as abusive [11].

Another challenge is that psychological violence is often characterized in different ways. For example, the conceptualization of “coercive control” can generally be understood in two ways: firstly, as an overall attempt to control one’s partner, in which IPV is a way of achieving control; secondly, as a subtype of IPV which is similar to—or a part of—the concept of psychological violence. The former is described in a structural perspective as more severe and gender-asymmetrical and is generally understood to be a representation of gender inequality [14–18]. The latter reflects a continuum of IPV from *psychological aggression* to more controlling behaviours constituting an assault [11]. Moreover, these are theoretical distinctions that have proven difficult for researchers and practitioners to conceptualize and apply.

The distinction between psychological violence and coercion is evident from the WHO, who define psychological violence (i.e. emotional or psychological abuse) such as “insults, belittling, constant humiliation, intimidation (e.g. destroying things), threats of harm, threats to take away children”, while controlling behaviour is defined as “isolating a person from family and friends; monitoring their movements; and restricting access to financial resources, employment, education or medical care” [1] (p. 1). The specific acts of violence separate these two definitions, while the overall psychological harm combines them. Likewise, the European Institute of Gender Equality (EIGE) states an overall definition of psychological violence, which can be understood from the perspective of caused harm: “Any act or behaviour which causes psychological harm to the partner or former partner. Psychological violence can take the form of, among others, coercion, defamation, a verbal insult or harassment”^19^ (p. 45).

These variations in terminology are inevitably reflected in the psychometric instruments developed to assess psychological violence (Table 1; identified by the Centers for Disease Control and Prevention and the National Unit [20] against IPV in Denmark, LUV [21]). Indeed, the varying use of subscales (e.g. #7 and #9) and differing definitions of psychological violence make the findings from these studies difficult to compare and stresses the need to evaluate how the effect on mental health is influenced by such variations. For example, some instruments assess psychological aggression (e.g. #10), while others measure more severe controlling behaviours (e.g. #4). Furthermore, other instruments focus less on the *act* of violence and more on the *effect* on the victim (e.g. #12). When measuring the effect of psychological violence on mental health, the difference between acts and effects can be crucial. Evidence demonstrates that some acts defined as psychologically violent (i.e. threats to kill/harm) have been found to significantly load on a physical violence factor, most likely due to the aspect of physical threat, which makes the psychological and physical aspect hard to differentiate [11]. Furthermore, a majority of these assessment tools make use of frequency scores that do not differentiate between the severity of items. As such, items of *psychological aggression* (i.e. being called “ugly” and “worthless”) are equated to severe controlling behaviour and threats (i.e. threats to kill or take away children). This exemplifies how important these distinctions are if we wish to understand the independent effect of psychological violence on mental health. Examining mental health while evaluating the instruments used to measure the phenomenon will help us understand whether severity, frequency or actual “type” of psychological violence produces the most harm on the victim’s mental health [11].

**Table 1:** Psychometric measures of psychological violence

#	Developer	Assessment	Characteristics	Target groups	Psychometrics
1	Shepard & Campbell [29] Copyright 1992	Abusive Behavior Inventory	30-item scale with 2 subscales that measure the frequency of physical and psychological abusive behaviours. The psychological abuse subscale includes 17 items.	Females with current or former intimate partners.	Internal consistency: Psychological abuse = 0.88–0.92. Evidence of convergent, discriminant, criterion and factorial validity
2	Hegarty, Sheehan, and Schonfeld [30]; Hegarty, Bush, and Sheehan [31] Copyright 1999	Composite Abuse Scale (CAS)	30-item scale with 4 subscales that measure severe combined abuse, emotional abuse, physical abuse and harassment. The emotional abuse subscale includes 11 items.	Females with current or former intimate partners for longer than one month.	Internal consistency: Emotional abuse = 0.93. Evidence of content, construct, criterion and factorial validity
3	Sullivan and Bybee [32]; Sullivan, Parisian, and Davidson [33]; O’Leary [34]	Index of Psychological Abuse	33-item scale that measures the degree to which assailants used ridicule, harassment, criticism, and emotional withdrawal.	Females in dating and marital relationships.	Internal consistency: 0.97
4	Rodenburg and Fantuzzo [35] Copyright 1993	Measure of Wife Abuse	60-item scale with 4 subscales that measure the frequency of physical, sexual, psychological and verbal abusive behaviours. The psychological abuse subscale includes 15 items and the verbal abuse subscale includes 14 items.	Females with current or former intimate partners.	Internal consistency: Total scale = 0.93 Verbal abuse = 0.83 Psychological abuse = 0.94 Evidence of convergent and factorial validity
5	Murphy and Hoover [36]; Murphy, Hoover, and Taft [37]	Multidimensional Measure of Emotional Abuse *MMEA*	28-item scale (reduced from 54 items) that measures restrictive engulfment, hostile withdrawal, denigration and dominance/intimidation.	College students reporting on current or past dating relationships.	Internal consistency: Restrictive engulfment = 0.85 Hostile withdrawal = 0.91 Denigration = 0.92 Dominance/intimidation = 0.91 Evidence of convergent and discriminant validity
6	Hudson [38] Copyright 1992	Partner Abuse Scale—Non-Physical	25-item scale that measures the magnitude of perceived nonphysical abuse received from a spouse or partner; 2 of the items assess sexual abuse.	Partners in dating, cohabiting and marital relationships.	Internal consistency: > 0.90. Evidence of content and factorial validity
7	Sackett and Saunders [39] Copyright 1999	Profile of Psychological Abuse	21-item scale that measures a wide variety of psychological abuse.	Abused females.	Internal consistency: Jealous control = 0.85 Ignore = 0.80 Ridicule traits = 0.79 Criticize behaviour = 0.75 Evidence of convergent and criterion validity
8	Tolman [40, 41]	Psychological Maltreatment of Women Inventory (PMWI).	58-item scale that measures psychological maltreatment of women by their male intimate partners.	Females in intimate relationships.	Internal consistency: Dominance/isolation = 0.95 Emotional/verbal = 0.93 Evidence of convergent, discriminant, criterion and factorial validity
9	Tolman [41]	Psychological Maltreatment of Women Inventory (PMWI)—Short Form	14-item scale that measures psychological maltreatment of women by their male intimate partners.	Females in intimate relationships.	Internal consistency: Dominance/Isolation = 0.88 Emotional/verbal = 0.92 Evidence of construct, convergent and discriminant validity
10	Straus, Hamby, Boney-McCoy, and Sugarman [42]; Straus, Hamby, and Warren [43] Copyright 2003	Revised Conflict Tactics Scales (CTS-2)	78-item scale that assesses both victimization and perpetration. The 39-item victimization scale includes 5 subscales that measure physical assault, psychological aggression, sexual coercion, negotiation and injury between partners. The psychological aggression subscale includes 8 items that assess verbal and symbolic acts that are intended to cause fear or psychological distress.	Partners in dating, cohabiting and marital relationships.	Internal consistency: Psychological aggression = 0.80 (Mechanic et al., 2000b); 0.82 (Lucente et al., 2001)
11	Foshee, Linder, Bauman et al. [44]; Foshee et al. [45]	Safe Dates— Psychological Abuse Victimization	14-item scale that measures psychological victimization in dating relationships.	Male and female students in grades 8-9.	Internal consistency: 0.91.
12	Smith, Earp, and DeVellis [46]; Smith, Smith, and Earp [47]; Smith, Thornton, DeVellis, Earp, and Coker [48] Copyright 2002	Women’s Experiences with Battering (WEB)	10-item scale that measures prevalence of the battering of women.	Females with current or former male intimate partners.	Internal consistency: 0.91–0.99 Evidence of convergent, discriminant, and critierion validity
13	*Sherin et al* [49] *Copyright* [50]	*Hurt*, *Insulted*, *Threatened with Harm and Screamed* (*HITS*) *Domestic Violence Screening Tool*	*4*-*item scale with one question on physical violence and three on psychological*: *insult*, *threat*, *and scream*. *Measured on 5*-*point Likert scale from* “*Never*” *to* “*Frequently*”.	*Both male and female victims of domestic violence*.	*Cronbach*’*s alpha 0*.*85*
14	*Swahnberg and Wijma* [51].	*The NorVold Abuse Questionnaire* (*NorAQ*)	*Three*-*item subscale of emotional abuse* (*mild*, *moderate and severe*; “*No*”, “*Yes*, *as a child < 18*”, “*Yes*, *as an adult* ≥ *18*”, “*Yes*, *as a child and an adult*”). “*Current suffering*” *from abuse measured on 11*-*point Likert from* “*0 = No Suffering*” *to* “*10 = Terrible Suffering*”.	*Female experiences of physical*, *sexual and emotional abuse*.	*Good validity and test*-*retest reliability*
15	Graham-Kevan and Archer [52]	*Controlling Behaviors Scale*-*Revised* (*CBS*-*R*)	*24*-*item behavioural scale with 5 subscales*; “*economic abuse*”, “*coercion and threats*”, “*intimidation*”, “*emotional abuse*”, *and* “*isolation*”. *Report on one*’*s own and partners behaviour on 5*-*point Likert scale from* “*0 = never*” *to* “*4 = always*”. *Either four sub*-*scores or one total*-*score*, *separately for self and partner*.	*Developed to compare across sample types for both male and female respondents*.	*Cronbach*’*s alpha = 0*.*86*.
16	*Follingstad*, *Coyne*, *and Gambone* [53]	*Follingstad Psychological Aggression Scale* - *FPAS*	*17*-*items each representing a category of psychological violence*. *Items are grouped in three subscales*: *mild*, *moderate and severe*. *Scored on Likert scale from 1*-*10 from* “*not psychological abuse at all*” *to* “*extreme psychological abuse*”.	*Victims of psychological abuse*.	*Internal consistency Cronbach*’*s alpha was 0*.*98* (*mild*, *moderate and severe items*: *0*.*92*, *0*.*95*, *and 0*.*96*).
17	*Dutton*, *Goodman and Schmidt* [54]	*Coercion in Intimate Partner Relationship Scale*.	*48*-*items with 9 subscales*; *Personal Activities/Appearances*, *support/social life/family*, *household*, *work/economic/resources*, *health*, *intimate relationship*, *legal*, *immigration*, *children/parenting*.	*Two separate sets of 48*-*items for both demands made by partner to respondent and by respondent made to partner*.	*Strong support for convergent validity and evidence of predictive validity*.
18	*Follingstad* [55]	*Measure on Psychologically Abusive Behaviors* (*MPAB*)	*14*-*items each representing a category of psychological violence* (*more severe than FPAS*); *Items are grouped in three subscales*; *mild*, *moderate and severe*. *Scored on Likert scale from 1–10 from* “*not a violation at all*” *to* “*strong violation*”. *Malignant intention incorporated in questions*.	*Usable with dating or married populations*, *cohabitating or non*-*cohabiting relationships*, *males or females*, *and heterosexual or homosexual couples*.	*Internal consistency 0*.*98* (*mild*, *moderate and severe items*: *0*.*94*, *0*.*94 and 0*.*94*).
19	*Rogers and Follingstad* [56]	*Global Perceived Harm* (*PH*)	*Eight*-*item scale measuring believed effect of partners psychological maltreatment on psychological*, *physical and/or daily functioning as well as negative perception of relationship and world in general*. *Scored on 5*-*point Likert scale from* “*a little*” *to* “*a lot*”.	*A scale reflecting impacts from women*’*s experience with battering and negative changes seen in oneself and one*’*s relationship*.	*Reliability statistic of 0*.*93*.
20	*Campbell*, *Campbell*, *King*, *Parker and Ryan* [57]	*Index of Spouse Abuse* (*ISA*-*NP*)	*The ISA is a 30*-*item abuse scale with a 19*-*item subscale of non*-*physical abuse measured on a 5*-*point Likert scale from* “*1 = never*” *to* (*5 = very frequently*). *Clinical cut*-*off score for non*-*physical is 25*.	*Female victims of physical and emotional abuse*.	*Alpha coefficient 0*.*95*.
21	Campbell et al. [58] Copyright 2004.	Revised Danger Assessment (DA)	Risk assessment for femicide; 20-items (both physical, sexual and psychological). Number of total “yes” answers.	Female victims of severe battering.	Sensitivity of R-DA ranged from 0.545 from extreme Danger level to 0.987 if increased danger was used (Mean sensitivity = 0.750 and specificity = 0.863).

Psychological/Emotional Victimization Scales adapted from Thompson, Basile, Hertz and Sitterle [20] with added information in italic writing by the authors (with inspiration from Oldrup, Andersen, Kjær, Nielsen, and von Rosen [21]).

In addition to problems with the conceptualization, a number of methodological challenges further characterize the field, e.g. sampling, design, scoring and gender bias [11]. Focusing on psychological aggression in lesbian, gay and bisexual individuals, Mason et al. [22] highlight the need for future research to clearly and consistently define psychological violence and separate it from other types of violence seeing that a more consistent definition will facilitate better comparisons across studies. The research group further stresses that scoring methods (e.g. frequency vs. dichotomous scoring) influence the magnitude of the effect size, which makes results difficult to compare. This challenge is further complicated by the use of self-administered questionnaires that may lack systematic development [22]. Moreover, Follingstad [11] emphasizes the need to differentiate between samples (i.e. dating relationships and marital or long-term cohabiting relationships), seeing that dating relationships are characterized by quantitatively and qualitatively less psychological violence. Finally, the majority of assessment tools are developed to specifically measure female victimization of psychological violence, despite male victimization being reported at equally high rates in some studies [7]. Although fewer studies have focused on the effects on mental health among male victims of psychological violence, studies indicate that they too present symptoms of anxiety, depression and sleep disturbances [13, 23].

The aim of the present systematic review is to build on existing knowledge [13] concerning the effect of psychological violence on mental health, while evaluating the psychometric instruments used to assess psychological violence about how they conceptualize the phenomenon. To this day, most studies on IPV and mental health have pooled scores of physical, psychological and sexual IPV in their reporting [2, 4], making a distinction of the individual effect of psychological violence difficult. When directly examining the effect of psychological violence, the lack of a clear and consistent definition of psychological violence has made results difficult to compare [13, 22]. Consequently, important information is lost. The present study therefore aims to consolidate knowledge on psychological violence by evaluating whether frequency, severity or actual “type” of psychological aggression is associated with the most harm on mental health [11]. Methodological challenges will be considered by conducting quality assessments of all included studies, and results will control for the presence of physical and sexual violence (i.e. severity, duration and frequency). When possible, mediating and moderating factors will be evaluated, as well as potential gender differences. Based on the results, a discussion on assessment tools and methodological challenges will provide the grounds for recommendations concerning future research.

To sum up, the aim of this systematic review and meta-analysis is fourfold: (1) to estimate the individual effect of psychological violence on mental health (e.g. PTSD, depression and anxiety); (2) to estimate whether frequency, severity or actual “type” of violence is associated with the most harm; (3) to investigate gender, sampling and cultural differences through moderation analyses; and (4) to discuss the somewhat vague terminology and methodological challenges.

## Methods and design

### Methods of review

The present protocol has been written in accordance with the Preferred Reporting Items for Systematic reviews and Meta-Analyses (PRISMA) guidelines and is presented in accordance with the PRISMA-P checklist (Additional file 1). The protocol has further been registered in PROSPERO (#CRD42018116026).

The systematic review will be conducted as an individual and dual process by two researchers (SD + DBH) in regard to screening, eligibility and inclusion. Screening will be done on a title basis, followed by an abstract and full-text basis. A third researcher (RK) from the team will be consulted to resolve issues regarding disagreement of eligibility and inclusion on a full-text basis. If sufficient data can be extracted, a random-effects meta-analysis will be conducted on the extracted data.

It is expected that not all studies report the recorded data on psychological violence, but rather cluster subtypes of IPV (i.e. physical, sexual and psychological violence). In such cases, the corresponding authors will be contacted and invited to share the raw data.

### Key definitions of the systematic review

#### Intimate partner violence

As proposed in a statistical definition by EIGE, IPV is defined as “any act of physical, sexual, psychological or economic violence that occurs between former or current spouses or partners, whether or not the perpetrator shares or has shared the same residence with the victim” [19] (p. 44). This definition has been proposed to aid the EU member states to collect and compare administrative data on violence against women in a standardized manner [19].

#### Psychological violence

A broad terminology for psychological violence is used in both scientific literature and in clinical practice (e.g. psychological violence, emotional abuse, coercion, psychological aggression). For clarity, this review will use the term *psychological violence* seeing that this definition links it directly to other types of IPV (e.g. physical or sexual violence), while emphasizing a core psychological aspect of harm in both the act of perpetration as well as the effect of victimization. The variation in terminology will shortly be addressed in the discussion.

In the current protocol, psychological violence will be defined by an overall definition combining that of both the WHO [1] (i.e. both emotional/psychological abuse and controlling behaviours) and the European Institute of Gender Equality [19]: “Any act or behaviour which causes psychological harm to the partner or former partner. Psychological violence can take the form of, among others, coercion, defamation, a verbal insult or harassment” [19] (p. 45), including belittling, constant humiliation, intimidation (e.g. destroying things), threats of harm, threats to take away children and/or isolating a person from family and friends; monitoring their movements; and restricting access to financial resources, employment, education or medical care [1] (p. 1). A broad definition will allow for studies using varying definitions to be included and will be assessed through subgroup analyses. The different definitions will be evaluated and discussed based on their effect on mental health.

### Mental health in this context

As mentioned above, it has previously been argued that psychological violence alone cannot be characterized as a trauma [11]. However, recent studies have identified an association between psychological violence and PTSD [13]. Based on these findings, the authors consider psychological violence a potential traumatic event and wish to further examine the relationship between psychological violence and PTSD. Other mental health consequences identified are based on the National Institute for Health and Care Excellence (NICE) guidelines and comorbid mental health problems of PTSD [24], i.e. depression, anxiety, alcohol or drug abuse, suicidality, sexual problems, sleep problems, problems with concentration, somatization and functional problems (e.g. social, educational, or occupational) as well as feelings of shame and guilt.

### Search method

A dual search will be conducted in the electronic databases PsycINFO, PubMed, EMBASE and Web of Science. Other methods used for identifying relevant research include reference checking and hand-searching of grey literature. Furthermore, the following scientific journals will be hand-searched: *Journal of Interpersonal Violence* and *Journal of Violence and Victims*.

#### Criteria for including studies

The review will include studies of psychological violence on mental health when controlling for other types of partner abuse. Hence, studies including an adult (≥ 18 years) population of victims of IPV (dating samples, national samples, clinical settings, etc.) that report on psychological violence specifically. Many studies are expected to include groups of comparison (e.g. non-abused or other types of abuse); however, comparisons are not required.

Furthermore, the review will only include peer-reviewed articles in English, German, Dutch or Scandinavian languages (i.e. Norwegian, Swedish or Danish).

#### Criteria for excluding studies

The review will exclude case studies, reviews, commentaries, editorials, letters to editorials, book chapters and other non-primary research articles.

### Search string

See Table 2 for the PsycINFO search string.

**Table 2:** Search String

	Search algorithm	Hits
1. Population	*Exp* exp Intimate Partner Violence/ OR exp COUPLES/ OR exp Partner Abuse/ OR exp Domestic Violence/ OR exp DYADS/ OR exp MARRIAGE/ OR exp HUSBANDS/ OR exp WIVES/ OR Wife OR *friend/ OR Girlfriend OR Boyfriend OR Dating OR Domestic OR Partner	
2. Exposure	*Keywords* Psychological Victimization OR Emotional Victimization OR Psychological Violence OR Psychological Abuse OR Psychological Assault OR Aggression OR Psychological Aggression OR Emotional Aggression OR Emotional Abuse OR Emotional Assault OR Emotional Violence OR Coercive Control OR Coercion OR Humiliation	
3. Measures/scales	*Keywords* Abusive behaviour Inventory OR (Composite Abuse Scale OR CAS) OR Index of Psychological Abuse OR Measure of Wife Abuse OR Multidimensional Measure of Emotional Abuse OR Partner Abuse Scale-Non Physical OR (Psychological Maltreatment of Women Inventory OR PMWI) OR (Revised Conflict Tactic Scale OR CTS2 OR CTS-2) OR Safe Dates - Psychological Abuse Victimization OR (Women’s Experiences with Battering OR WEB) OR (Tool for Intimate Violence Screening OR HITS) OR (NorVold Abuse Questionnaire OR NorAQ) OR (Controlling Behaviors Scale-Revised OR CBS-R) OR (Follingstad Psychological Aggression Scale OR FPAS) OR Yllo’s Controlling Behavior Questions OR Coercion in Intimate Partner Relationship Scale OR (Measure on Psychologically Abusive Behaviors OR MPAB) OR (Global Perceived Harm OR PH) OR (Index of Spouse Abuse OR ISA-NP) OR (Danger Assessment OR DA)	
4.	*2 OR 3*	
5. Outcome (mental health)	*Exp and keywords* exp Mental Health/ OR exp Emotional Trauma/ OR exp COMORBIDITY/ OR exp Posttraumatic Stress Disorder/ OR exp MAJOR DEPRESSION/ OR exp “DEPRESSION (EMOTION)”/ OR exp ANXIETY DISORDERS/ OR exp ANXIETY/ OR exp ALCOHOL ABUSE/ OR exp DRUG ABUSE/ OR exp ATTEMPTED SUICIDE/ OR exp SUICIDE/ OR Mental Health.mp. OR Emotional Trauma.mp. OR COMORBIDITY.mp. OR COMOR*.mp. OR Posttraumatic Stress Disorder.mp. OR Post-traumatic stress.mp. OR PTSD.mp. OR Posttraumatic stress symptoms.mp. OR Post-traumatic stress.mp. OR PTSS.mp. OR DEPRESSION.mp. OR Major Depression.mp. OR Depress*.mp. OR ANXIETY.mp. OR Anxiety DISORDERS.mp. OR Anxie*.mp. OR Panic*.mp. OR Phobia.mp. OR Social Anxie*.mp. OR Substance Abuse.mp. OR ALCOHOL ABUSE.mp. OR DRUG ABUSE SUICIDE.mp. OR ATTEMPTED SUICIDE.mp. OR Suicidal.mp. OR Shame.mp. OR Guilt.mp. OR Reduced Libido.mp. OR Sexual Problems.mp. OR Social Functioning.mp. OR Educational Functioning.mp. OR Occupational Functioning.mp. OR Sleep Problems.mp. OR Sleep Disorders.mp. OR Concentration Problems.mp. OR Job Loss.mp. OR Social Withdrawal.mp. OR Social Isolation.mp. OR Somatization.mp. OR Somatic Complaints.mp. OR Chronic Pain.mp. OR Pain.mp. OR Poor Health.mp. OR Medical Problems.mp. [mp=title, abstract, heading word, table of contents, key concepts, original title, tests & measures]	
6.	*1 AND 4 AND 5*	

### Main outcome of interest

To investigate the independent effect of psychological violence on mental health. A meta-analysis will be performed to estimate the effect of psychological violence on PTSD, depression and anxiety. A narrative summary will present all related mental health problems as defined by the NICE guidelines [24].

Secondary outcomes of interest include the following:

- How does “type” of psychological violence affect mental health?
- How does frequency and severity affect mental health outcomes? (e.g. high frequency and/or low severity or low frequency and/or high severity).
- Are there potential gender differences in mental health consequences with regard to psychological violence?
- Will controlling for previous trauma affect the association between psychological violence and mental health?
- Does sample population differ in mental health consequences? (e.g. dating samples vs. treatment samples)

Finally, the systematic review aims to evaluate included psychometric tools measuring psychological violence and how potential variations in the conceptualization of psychological violence affects results.

### Data extraction

Data will be extracted with help from Endnote and Covidence. Meta-analyses will be conducted using the programming language R. The authors will design a data extraction form. The form will include authors, year, sample size, population, country, age, gender, design, IPV assessment tool(s), mental health assessment, primary outcome (effect size), secondary outcome(s), timeframe of assessment (lifetime or specified), scoring method(s), previous trauma and previous mental health problems. Data will be extracted by one reviewer (SD), and independently cross-checked by another reviewer (DBH). Inconsistencies in data extraction will be resolved between the reviewers by referring to the source study until a consensus is reached.

#### Quality assessment

The “Quality Assessment Tool for Quantitative Studies” developed by the Effective Public Health Practice Project [25] will be used to assess the quality of the included studies. This is in line with previous research evaluating IPV psychometric tools [26]. Assessment will be based on six components: (1) selection bias, (2) study design, (3) confounders, (4) blinding, (5) data collection methods and (6) withdrawals and dropouts [24, 25]. Two researchers (SD and DBH) will classify studies on three levels: weak, moderate and strong. If classifications are inconsistent a third researcher (RK) will be involved and classification will be discussed until consensus is reached. If possible, moderation analyses will compare studies of strong vs. weak quality.

### Plan for data synthesis

As demonstrated above, studies on psychological violence are quite heterogeneous in regard to conceptualization, psychometrics, sampling, design, scoring, and so forth. Therefore, we will perform a random-effects meta-analysis, because we expect high heterogeneity in the included studies. The random-effects meta-analysis assumes variance in effect across studies due to real differences in effect as well as by chance. The meta-analysis will help estimate the common effect of psychological violence on mental health (i.e. PTSD, depression and anxiety) by synthesizing individual results. If possible, moderation analyses will compare studies according to quality assessment and varying samples. The *I*^2^ statistic will be used to test for heterogeneity, and as suggested, an *I*^2^ statistic above 75% implies considerable heterogeneity, while an *I*^2^ statistic below 40% is not considered to be a concern [27].

The included studies are expected to report effect sizes of varying types (i.e. correlation, regression, mean differences and association of categorical variables, e.g. odds ratio). For data synthesis, the reported effect sizes will be recoded into the same type of effect size using the programming language R. For this meta-analysis, we expect to perform a stepwise analysis according to the outcome of interest. For the main outcome of interest, as well as subgroup analyses, the meta-analysis will be conducted with effect sizes based on correlations. Additionally, the impact of the duration and frequency of psychological violence will be estimated by using meta-regression. The Metafor-package for the programming language R will be applied to conduct the meta-analysis [28].

## Discussion

The present review will build on existing knowledge by statistically synthesizing results on the effect of psychological violence on mental health. The review will help consolidate knowledge on psychological violence by evaluating whether frequency, severity or actual “type” of psychological violence produces the most harm. This will strengthen our knowledge on psychological violence, and how best to assess and conceptualize the phenomenon.

While conducting the systematic review, we expect to encounter several limitations. The varying terminology and definitions of psychological violence will make results difficult to compare, just as the many and varying psychometrics tools will (Table 1). This is further complicated by the fact that many studies are likely to not even use validated instruments but instead base their results on a few self-constructed items. The review will aim to overcome these limitations by thorough quality assessment of the included studies with help from the Quality Assessment Tool for Quantitative Studies [25] and by performing relevant subgroup analyses. This does not only apply to psychological violence, but also mental health definitions and the psychometric instruments used to assess symptomatology. By critically examining the applied definitions and terminology as well as methodological challenges (e.g. sampling, design, scoring and gender bias) the review will serve as a status quo of the field and make grounds for future recommendations.

Finally, the systematic review is expected to have several clinical implications. We expect the meta-analysis to deepen our understanding of the different subtypes of psychological violence and how they independently interact with mental health outcomes. Likewise, we expect to give clarity on psychological violence and whether it should be understood as a traumatic event equal to other types of abuse (e.g. physical or sexual violence). Developing both preventive efforts and treatment programmes such information is important if we wish to directly target the needs of those affected by psychological violence and raise awareness to encourage both victims and perpetrators to seek help.

In research, this systematic review is expected to inspire researchers to clearly and consistently define psychological violence while carefully considering the psychometrics used to measure the phenomenon, as well as other methodological challenges. Moreover, we expect to get clarity on any potential gender bias.

On a final note, this work will hopefully inspire others to conduct similar research on children who grow up as either witnesses or victims of psychological violence.

## Additional file

Additional file 1: PRISMA-P 2015 Checklist (DOCX 30 kb)

## Data Availability

The datasets used and/or analysed during the current study are available from the corresponding author on reasonable request.
